# Unlocking NuriPep 1653 From Common Pea Protein: A Potent Antimicrobial Peptide to Tackle a Pan-Drug Resistant *Acinetobacter baumannii*

**DOI:** 10.3389/fmicb.2019.02086

**Published:** 2019-09-18

**Authors:** Niamh Maire Mohan, Amine Zorgani, Gael Jalowicki, Alish Kerr, Nora Khaldi, Marta Martins

**Affiliations:** ^1^Department of Microbiology, Moyne Institute of Preventive Medicine, School of Genetics and Microbiology, Trinity College Dublin, University of Dublin, Dublin, Ireland; ^2^Nuritas Limited, Dublin, Ireland

**Keywords:** antimicrobial peptides, multidrug resistance, *Acinetobacter baumannii*, data mining, hydrolyzed proteins, novel antimicrobials

## Abstract

While the antibiotic era has come and gone, antimicrobial peptides (AMPs) hold promise as novel therapies to treat multidrug resistant (MDR) pathogens in an age where the threat of multidrug resistance escalates worldwide. Here, we report the bactericidal properties of NuriPep 1653, a novel 22 mer and non-modified peptide. NuriPep 1653 was identified within the sequence of the non-antimicrobial P54 protein, which is involved in nutrient reservoir activity in *Pisum sativum*. Total bacterial clearance of *Acinetobacter baumannii* cells (1 × 10^8^ cells/mL) was observed using only 4 × MIC (48 μg/mL) of NuriPep 1653 after just 20 min of treatment. We uncovered a synergistic interaction between NuriPep 1653 and another antimicrobial peptide, colistin. The MIC of NuriPep 1653 and colistin dropped from 12 and 8 μg/mL to 2 and 1 μg/mL, respectively, when they were combined. NuriPep 1653 exhibits no cytotoxicity in different human cell lines and has a low propensity to induce bacterial resistance in a colistin resistant clinical isolate of *A. baumannii*. The existence of these peptides embedded in proteins unearths potentially new classes of antimicrobials with activity against clinically relevant pathogens. Our findings push the boundaries of traditional peptide discovery and represent a leading edge for natural bioactive compounds which may have a common existence in nature but remain unexposed.

## Introduction

As we descend deeper into a post antibiotic era, the incidence of multidrug resistance extends to epidemic proportions. A significant threat, as recognized by the World Health Organisation (WHO) as a primary critical priority concern, is carbapenem-resistant *Acinetobacter baumannii* (World Health Organisation [WHO], 2017; Tacconelli et al., 2018). *A. baumannii* is an opportunistic, coccobacillus, Gram-negative bacterium. The importance of this nosocomial pathogen is escalating based on its ability to persist on artificial surfaces for extended periods as well as its propensity to acquire resistance to antibiotics (Ahmed et al., 2016). It is often life-threatening for critically ill, immunocompromised patients and is a major factor in complicating the treatment and rehabilitation of injured soldiers (Ahmed et al., 2016). For many years, carbapenems were considered as the standard therapeutic agents for multidrug resistant (MDR) *A. baumannii* infections, however, their effectiveness is waning as the incidence of extensively drug resistant (XDR) pathogens continues to rise (Kim et al., 2014; Qureshi et al., 2015). Despite being abandoned for use in the 1970’s, due to significant renal and neurological toxicity, bacterially derived lipopeptides, such as colistin, are now frequently used as a salvage treatment for XDR *A. baumannii* infections (Spapen et al., 2011; Pogue et al., 2015). Unfortunately, increased reliance on this last-line therapy has also spurred resistance and has fueled the surging progression from XDR to pan drug resistant (PDR) *A. baumannii* posing major complications for modern healthcare (Qureshi et al., 2015; Subramani et al., 2017; Tacconelli et al., 2018).

A recent revival in the exploration of antimicrobial peptides (AMPs) as antibiotic alternatives has come as a consequence of the dwindling armory of effective antimicrobial drugs and subsequent rise in resistance. The revival has transpired based on two critical attributes, their low propensity for inducing resistance and their broad spectrum activity based on a general mechanism of action (Meher et al., 2017). These attributes come as a result of the two major mechanisms associated to cationic peptides including direct bacterial killing, which is either membrane or non-membrane targeting, and immune modulation. It is well described in the literature that the majority of AMPs display the former, where the peptide is attracted to the cell membrane by electrostatic interactions between the positively charged residues of the peptide and the negatively charged components in the bacterial cell membrane (Yeaman and Yount, 2003; Kumar et al., 2018). The general mechanisms of action described for AMPs means that they are often active against both antibiotic susceptible and resistant bacteria, which places them competitively among new antimicrobial solutions for the pharmaceutical sector.

The majority of AMPs discovered to date are non-ribosomal secondary metabolites assembled by enzymes in mammals, insects and bacteria. Until recently, very few had been exploited commercially as antimicrobials based on their toxicity, complex structures, and high production costs. A largely unexplored class of peptides, which may have an improved safety profile based on their source, are those found within regions of proteins (Schaafsma, 2009). While the reason for the presence of these sequences, entrenched in proteins, is largely undetermined, it provides a new avenue of discovery for anti-infectives and peptides with other bioactivities. Recently, human pepsinogen A3-derived peptides were shown to exhibit potent antimicrobial functions in skin infection models whilst showing no toxicity to human cells (Pane et al., 2018). In another study, lactoferricin, a non-toxic AMP released from the milk glycoprotein, lactoferrin, post digestion is described (Bruni et al., 2016). In the case of milk, the release of functional peptides from proteins occurs in the mammary gland, were complex proteases pre-digest the proteins into bioactive peptide fragments for neonatal health (Dallas and German, 2017). While further investigation is needed, bioactive peptides within phyto-proteins may confer specific health benefits or protection once digested in a similar way to milk, or else may have a role in the life cycle of the plant. In this study, we explore if we can apply the same rationale for AMPs discovery in milk proteins, to plants, by finding peptides within edible plant proteins which exhibit potent antimicrobial effects against PDR *A. baumannii*. Here, we describe NuriPep 1653, a novel, 22-mer peptide with the sequence VRGLAPKKSLWPFGGPFKSPFN, which was discovered in the region between residues 271–292 of the P54 nutrient reservoir protein of *Pisum sativum*. NuriPep 1653 was among one of the peptides in the library which were released after hydrolysis and was chosen based on a combination of features which we identified as most associated in conferring antimicrobial activity. These 20 features are listed in Supplementary Table S1. We provide an insight into NuriPep 1653’s potent bactericidal activity against carbapenem and colistin resistant *A. baumannii*. We also show its limited toxicity, adjuvant properties with colistin and its reduced likelihood to induce resistance. This study highlights the discovery of a novel AMP sequence from within a plant protein which may have important therapeutic significance against MDR, XDR, and PDR strains.

## Materials and Methods

### Deciphering Features Conferring Antimicrobial Activity in Peptides

**FIGURE 1:**
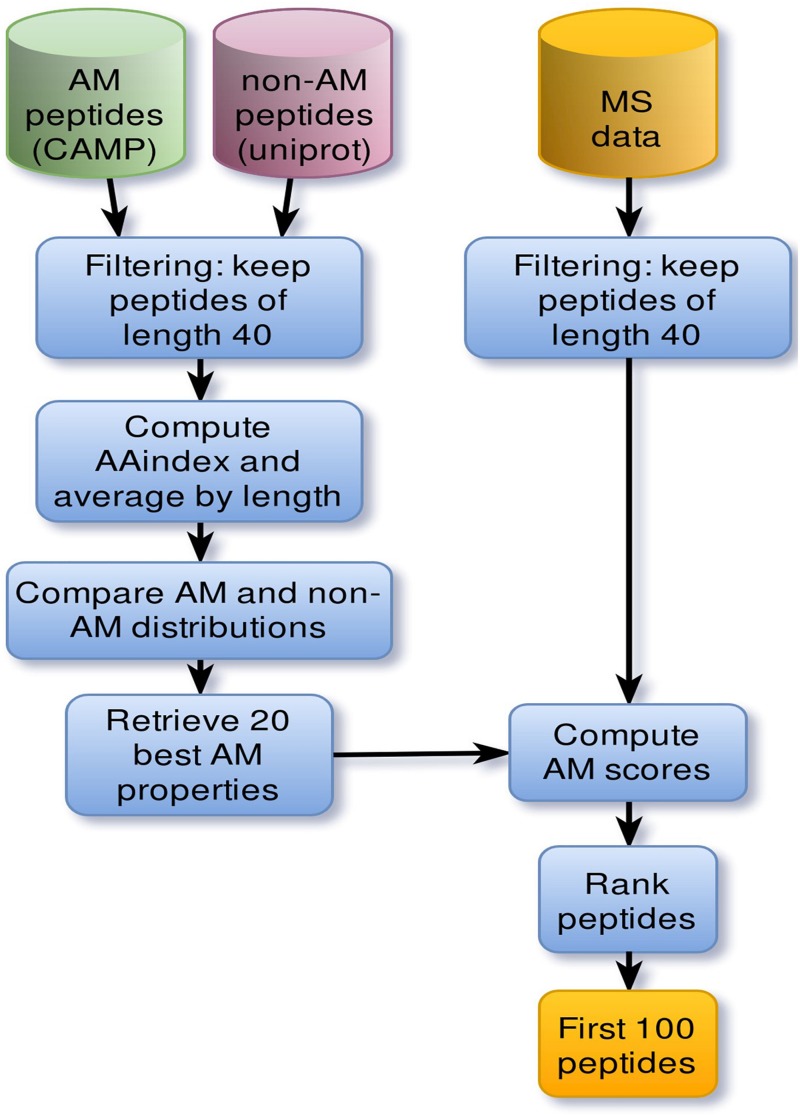
Main steps of the pipeline that led to the discovery of NuriPep 1653. Computational steps involved in the discovery of antimicrobial related properties and AMPs. Cylinders correspond to datasets. First positive (green) and negative (red) datasets were extracted from CAMP and Uniprot databases, respectively. Sequences whose length exceed 40 amino acids were discarded based on the cut-off length for peptides. Sequence physiochemical properties were computed based on individual amino acid scores retrieved from AAindex. Those scores were then normalized by sequence length. Comparison of property distributions yielded 20 main antimicrobial properties which were subsequently used to rank mass spectrometry (MS) peptides. The resulting set of 100 peptides were then manually curated.

The discovery pipeline is shown in Figure 1. AMPs were collected from the Collection of Anti-Microbial Peptides (CAMP^1^) database (Waghu et al., 2016). To focus on relatively short peptides, active sequences were filtered by a length threshold of 40 for a total of 1957 sequences. A set of 5000 non-AMPs whose length does not exceed 40 amino-acids was also extracted from Uniprot^2^. Only sequences containing standard amino acids were considered. Individual physicochemical properties of amino acids were pre-selected from curated cationic AMPs. For each physicochemical property, values were summed across each peptide sequence and then averaged by sequence length. To evaluate which properties were relevant to antimicrobial activity, property distributions from both active and negative datasets were compared using the non-parametric Kolmogorov–Smirnov test. Sequence scores were then ranked, and the top 20 properties kept as the most significant to antimicrobial activity.

### Peptides, Antibiotics and Reagents

The peptide sequence was synthesized by GenScript (Piscataway, NJ, United States) using solid-phase 9-fluorenylmethoxycarbonyl (Fmoc) chemistry and purified to a purity of 95–99% using reverse-phase high-performance liquid chromatography (HPLC). Peptide mass was confirmed by mass spectrometry (MS). Mueller-Hinton broth (MHB) and Mueller-Hinton agar (MHA) Oxoid brand were purchased from Sparks Lab Suppliers, Ireland. All antibiotics powders and discs were purchased from Sparks Lab suppliers. The LIVE/DEAD *Bac*Light kit was supplied by Molecular Probes (Invitrogen, Ireland). All 96 well plates used in this study were made with Polypropylene.

### Bacterial Culture Preparation and Growth Conditions

*A. baumannii* resistant (ColRAB) and susceptible (colSAB) (ATCC 19606) to colistin were kindly donated from the Institute of Hygiene and Tropical Medicine, Universidade Nova de Lisboa, Lisbon, Portugal. ColRAB was isolated from a bronchial secretion of a patient presenting with pneumonia and a urinary tract infection post bladder reconstruction (Machado et al., 2018). *Pseudomonas aeruginosa* ATCC 27853, *Escherichia coli* ATCC 25922, *Salmonella* Typhimurium ATCC 14028S, *Listeria monocytogenes* NCTC 11994, *Enterobacter aerogenes* ATCC 13048, and *Staphylococcus aureus* ATCC 25923 were gifted from the School of Food Science and Environmental health, Dublin Institute of Technology, Dublin. *Burkholderia cepacia* NCTC 10744 and *Stenotrophomonas maltophilia* NCTC 10257 were purchased from Public Health England (United Kingdom). *Klebsiella pneumoniae* isolate 50183 was kindly donated from St. James’ hospital, Dublin. Cultures were maintained at −80°C in 15% glycerol. All bacteria were grown in MHB. Cultures were prepared by inoculating 10 mL of the selected media with bacteria and incubating overnight for 18 h at 37°C. A subculture was prepared from the overnight bacterial suspension by diluting it to an optical density at 600 nm (OD_600_) of ∼ 0.05 in fresh media and re-incubating under optimal conditions for each bacterium for 2–3 h until logarithmic phase was reached (OD_600_ ∼ 0.5). Bacterial cultures were then adjusted to the desired concentration for assay in phosphate buffer without salt (PBNS).

### Disk Diffusion Method

The antibiotic susceptibility profile of the clinical isolate of *A. baumannii* was re-checked against the values previously reported by Machado et al. (2018). This was conducted using the Kirby Bauer disk diffusion method as per CLSI guidelines (Cinical Laboratory Standards Institute [CLSI], 2013). Briefly, agar plates were lawned with bacteria diluted to OD_600_ ∼ 0.1. Antibiotic disks were placed onto the agar surface and allowed to incubate overnight at 37°C. The zone of inhibition (ZOI) produced by the antibiotic was then used as a measure of susceptibility or resistance of the bacteria to the antibiotic according to the breakpoints outlined in the EUCAST guidelines (The European Committee on Antimicrobial Susceptibility Testing [EUCAST], 2019).

### Antimicrobial Activity Assays

#### Broth Microdilution Method

Minimum inhibitory and minimum bactericidal concentrations (MIC/MBC) were determined aerobically in a 96 well plate as previously described (Cinical Laboratory Standards Institute [CLSI], 2013). This method was used to determine the antibiotic susceptibility profile of the bacteria to antibiotics only, as an alternative method, described below, was used in the case of the peptides. Briefly, bacterial strains were grown aerobically shaking to mid exponential phase at 37°C in 5 mL of appropriate broth, depending on the strain. The compounds were then serially diluted to the desired concentrations in the microtiter plate to a final volume of 100 μL. Cultures were diluted in PBNS to a final concentration of 2.0 × 10^4^ and 4.5 × 10^5^ cells/mL and added to each well of the plate, except the negative control containing only media. The MIC was determined as the lowest concentration inhibiting bacterial growth after incubation at 37°C for 18 h and the MBC was defined as the lowest concentration to completely kill the bacteria. The MBC was performed using a pin replicator to transfer 1 μL from every well of the MIC plate into the corresponding wells of a compound free plate containing only 100 μL of media. The plate was then incubated at 37°C for 18 h. The MBC was determined as the concentration showing no growth at all (determined either by eye or by reading in a spectrophotometer at 600 nm) indicating complete cell death.

#### Complete Elimination (CE) Drop Plate Method

The CE method with PBNS buffer was primarily used as the antimicrobial susceptibility testing method for peptides used in this study. It should be mentioned that the CE assay was performed in various media including cation-adjusted Mueller Hinton broth, Nutrient Broth (NB), Brain Heart Infusion Broth (BHI), and Roswell Park Memorial Institute (RPMI), however, the ion content in all the above-mentioned media, interfered with the activity of the peptide. Hence, PBNS was used in order to perform the experiments included in this paper and to solely study the action of the peptide on the bacteria and avoid any interference of cations within the test media or buffer on the bioactivity of the peptides. The CE concentrations in the above-mentioned media are highlighted in Supplementary Table S2. Travis et al. (2000) and Farkas et al. (2018) explain the modified buffers they used in order to avoid ion interference. The CE assay was performed by the method outlined by Chen et al. (2003) with minor modifications. Briefly, bacteria were grown overnight and sub-cultured to mid exponential phase. PBNS was used to make serial dilutions of bacteria to a concentration of between 1.0 × 10^4^ and 1.0 × 10^5^ cells/mL. The appropriate volume of peptide was added to the wells at a final volume of 100 μL. As a control, PBNS was added to the bacterial suspensions without any peptide. The microtiter plate was incubated for 90 min at 37°C. Post challenge, 10 μL from each well was spotted onto appropriate media, depending on the strain. This was conducted in triplicate on three separate days. The spots were left to dry, and the plates were incubated inverted. The first concentration to inhibit growth across all bacterial dilutions was determined as the CE concentration. Surviving colonies at sub-CE concentrations were counted after 18 h incubation at 37°C. The reduction in colony forming units (CFU) was assessed relative to the untreated control and the log reduction induced by peptide treatment was calculated. All antimicrobial activity assays were performed in triplicate and on independent days.

### Synergy Studies and Measurement of Fractional Inhibitory Concentration

Synergy studies were conducted using the above described CE drop plate method, however, two peptides were mixed in a series of concentration combinations using PBNS as a diluent before bacterial inoculation and subsequent incubation. The highest concentration combination tested was the individual CE concentrations of NuriPep 1653 and colistin, which were 12 and 8 μg/mL, respectively. The amounts of each compound were then reduced sequentially in various proportions until the lowest possible active combination was identified.

Traditionally, the fractional inhibitory concentration (FIC) in a drug mixture is defined as the sum of the FICs (ΣFIC) and is calculated with the equation;

$$\Sigma \,\mathrm{FIC}={\mathrm{FIC}}_{\text{A}}+{\mathrm{FIC}}_{\text{B}}=\left({\mathrm{CE}}_{\text{A}}^{\text{comb}}/{\mathrm{CE}}_{\text{A}}\right)+\left({\mathrm{CE}}_{\text{B}}^{\text{comb}}/{\mathrm{CE}}_{\text{B}}\right)$$

Where CE_A_ and CE_B_ are the killing concentrations of individual peptides A and B, respectively, and CE^comb^_A_ and CE^comb^_B_ are the killing concentrations of the peptides in combination. In this work, we used a similar concept and analyzed the ratios of FIC_A_ and FIC_B_. Synergy was interpreted as a FIC of =0.5; antagonism as a FIC of >4.0 and no interaction as a FIC >0.5–4.0 (Odds, 2003).

### Valence Sensitivity Study

A variation of the CE drop plate method was used to determine valence sensitivity of the peptide. The concentration of NuriPep 1653 required to kill bacteria diluted in NaCl or CaCl_2_ at concentrations ranging from 0 to 150 mM was determined (Menzel et al., 2016).

### Kinetics of Antimicrobial Activity

The killing kinetics, or time kill assay was performed according to a previously published standard protocol (Lorian, 2005). The experiment was performed in duplicate. Bacterial cells from mid exponential growth were collected, washed and adjusted to a final density of 1.0 × 10^7^ to 1.0 × 10^8^ cells/mL in PBNS buffer. The cells were incubated at 37°C with different concentrations (12, 48, and 192 μg/mL) of NuriPep 1653 to a final volume of 2 mL. At T10, 20, 30, 60, and 120 mins, 0.1 mL of the culture was removed, diluted appropriately in PBNS and spread plated on MHA. The CFU/mL at each timepoint was calculated after overnight incubation at 37°C. Cells treated with PBNS alone were used as the control.

### Membrane Damage of Bacterial Cells

#### Cell Viability and Membrane Permeabilization Assessed by Flow Cytometry

The Live/Dead *Bac*Light viability kit was used as described by Joshi et al. (2010) with minor modifications. *A. baumannii* cells were collected in mid exponential phase, washed three times with PBNS, and resuspended at a final concentration of 1 × 10^6^ cells/mL in the same buffer. This was followed by addition of NuriPep 1653 at the CE concentrations (12 μg/mL) and incubation at 37°C for 90 min. Cells treated with PBNS were used as the live control and cells heat treated for 60 min at 90°C served as the “dead” control (maximum of fluorescence). These samples were used to establish the initial gate between live and dead populations (data not shown). All samples were prepared to a final volume of 1 mL before the addition of 6 μL of a premixed solution containing a 1:1 ratio of SYTO9 (3.34 mM), a green-fluorescent nucleic acid stain and propidium iodide (PI) (20 mM), a red-fluorescent nucleic acid stain. Once the dyes were added, samples were incubated for 15 min at room temperature in the dark. Flow cytometry analysis was conducted using the Accuri C6 Flow Cytometer. For each sample 50,000 cells were analyzed. The height of the forward scatter (FSC) and side scatter (SSC) of stained cells was analyzed using 488/530 emission and excitation filters. Data was analyzed by C Flow Plus software (Becton Dickinson, San Jose, CA, United States).

#### Scanning Electron Microscopy

Suspensions of *A. baumannii* were grown in MHB until mid-exponential phase before being washed three times with PBNS and resuspended at an OD_600_ of ∼ 0.5. The CE concentrations of NuriPep 1653 against this bacterial density was determined (32 μg/mL) and used to treat the cells for 90 min. After incubation, the cells were pelleted *via* centrifugation at 14,000 rpm for 10 min and then washed with PBNS. This process was repeated three times with the final re-suspension done using glutaraldehyde (2.5% w/v) diluted in PBNS to fix the cells. The fixed cells were dehydrated in a hexamethyldisilazane (HDMS) gradient at 10, 30, 50, 70, 90, and 100% before a final overnight drying *via* evaporation. Finally, the samples were mounted onto stubs and sputter coated with gold. The cells were imaged in the Core Imaging facilities in the Conway Institute, University College Dublin (Dublin, Ireland) using a Hitachi Scanning Electron Microscope (Hitachi High Technologies Europe) at a voltage of 5.0 kV.

### *In vitro* Resistance Study

This assay was conducted as outlined by Ge et al. (1999) with some modifications. Briefly, the *in vitro* passage study involved exposing bacteria diluted to 1 × 10^4^ cells/mL in PBNS to NuriPep 1653 at one-half of the previously established bactericidal concentration. After 90 min of incubation at 37°C, 10 μL was spotted in triplicate on MHA and incubated again overnight. Post-incubation, surviving colonies were collected, diluted in PBNS and adjusted to 1 × 10^4^ cells/mL before repeated treatment with the sub-killing concentration of NuriPep 1653. The CE concentration was determined prior to the initiation of the study, after the 4th, 7th, 11th, and 14th passages. If the CE concentration after the fourth passage was greater than the original killing concentration, then for passages 5, 6, and 7, the amount of peptide used during the bacterial challenge was increased to one-half of that new CE concentration. This was continued over a 14-day course. Magainin and colistin were used as controls for the *in vitro* resistance study. This assay was performed in independent duplicates.

### Toxicity Assay

#### *Ex vivo* Viability Assay

Cellular viability was assessed in human THP-1 macrophages by a colorimetric assay that makes use of thiazolyl blue tetrazolium bromide (MTT, Sigma) (Silva et al., 2016). Cells were grown in RPMI medium supplemented with 10% of heat inactivated fetal bovine serum (FBS), 1% L-glutamine and 1% penicillin-streptomycin. At 90% confluence, cells were centrifuged and diluted to a 1:5 ratio with complete RPMI. The monocytes were differentiated into macrophages using 10 mM phorbol 12-myristate 13-acetate (PMA) before being seeded in a 96 well plate at a density of 1.0 × 10^4^ cells/well and incubated in the presence of 5% CO_2_ for 72 h at 37°C. Following this, cells were treated with increasing concentrations (0.05, 0.5, 5, 50, and 500 μg/mL) of NuriPep 1653, magainin 2 or colistin before re-incubation for a further 24 h. Post-incubation, 110 μL of MTT was added to each well at a final concentration of 500 μg/mL and incubated at 5% CO_2_ for 2 h at 37°C. Formazan crystals were dissolved by the addition of 100 μL 100% Dimethyl sulfoxide (DMSO) and gentle shaking for 5 min at room temperature, in the dark. The absorbance was measured at 570 nm in a microplate spectrophotometer (Synergy H1, Biotek). Maximum toxicity was determined by cells incubated with 100% DMSO. Cell viability was calculated as a per cent of a control treated with PBNS. Three technical replicates were performed each day on three independent days.

### Hemolytic Assay

The effects of NuriPep 1653 on sheep red blood cells (RBCs) was evaluated by a hemolysis assay (Lu et al., 2016). In brief, 100 μL of fresh peripheral blood from a healthy sheep was added with 4 μL of heparin and centrifuged at 25°C for 15 min at 2000 rpm. The RBCs were washed and resuspended three times in PBS and before being prepared as a 2% suspension. The RBCs were seeded in a 96 well plate along with increasing concentrations of the peptide to a final volume of 100 μL. Triton X-100 was used as a positive control (for lysis) and PBS used as the negative control (no lysis). Post-incubation at 37°C for 90 min, the samples were centrifuged at 2000 rpm for 10 min and the absorbance was measured at 540 nm in a microplate spectrophotometer (Synergy H1, Biotek). The degree of hemolysis was calculated according to the following: % of hemolysis = [(Sample absorbance – negative control)/(positive control – negative control)] × 100%.

## Results

### Antibiotic Susceptibility Profile of *A. baumannii* Strains

*A. baumannii* ATCC 19606 was susceptible to 7 of the 8 antibiotic classes tested (Table 1). Based on its susceptibility to colistin, it will be denoted as colSAB. Conversely, the clinical isolate showed resistance to all the antibiotics tested, including the last line lipopeptide, colistin, defining it as a PDR isolate which will be referred in this study as colRAB. Our results were in line with previously reported findings (Machado et al., 2018).

**TABLE 1:** Antibiotic resistance profile of colRAB and colSAB.

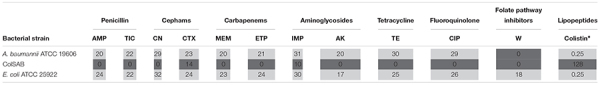

*The values refer to the diameter of a zone of inhibition produced by the antibiotic, measured in millimeters. Based on this value, the susceptibility profile to the antibiotics are grouped as defined by the CLSI guidelines as light gray = susceptible and dark gray = resistant. AMP, Ampicillin; TIC, Ticarcillin; CN, Cefotetan. CXT, Cefotaxime; MEM, Meropenem; ETP, Ertapenem; IMP, Imipenem; AK, Amikacin; TE, Tetracycline; CIP, Ciprofloxacin; W, Trimethoprim, and Colistin. ^∗^The antibiotic susceptibility profile of colistin was determined *via* broth microdilution method. *E. coli* ATCC 25922 was used as a reference quality control strain as per CLSI recommendations.*

### Unlocking and Identifying Potent Peptide Sequences From Vegetable/Legume Proteins

**FIGURE 2:**
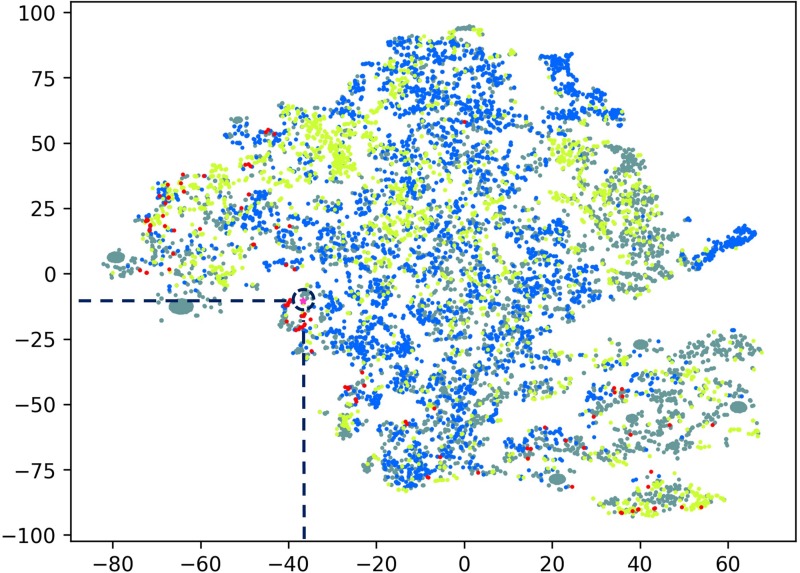
T-distributed Stochastic Neighbor Embedding (t-SNE) plot comprising all peptide data used in this study. Peptide sequences were transformed using all physiochemical features, averaged by peptide length. Gray: non-active peptides; yellow: active peptides; blue: peptides from mass spectrometry; red: top active peptides; Pink star: NuriPep 1653 (identified with a navy blue broken circle).

**FIGURE 3:**
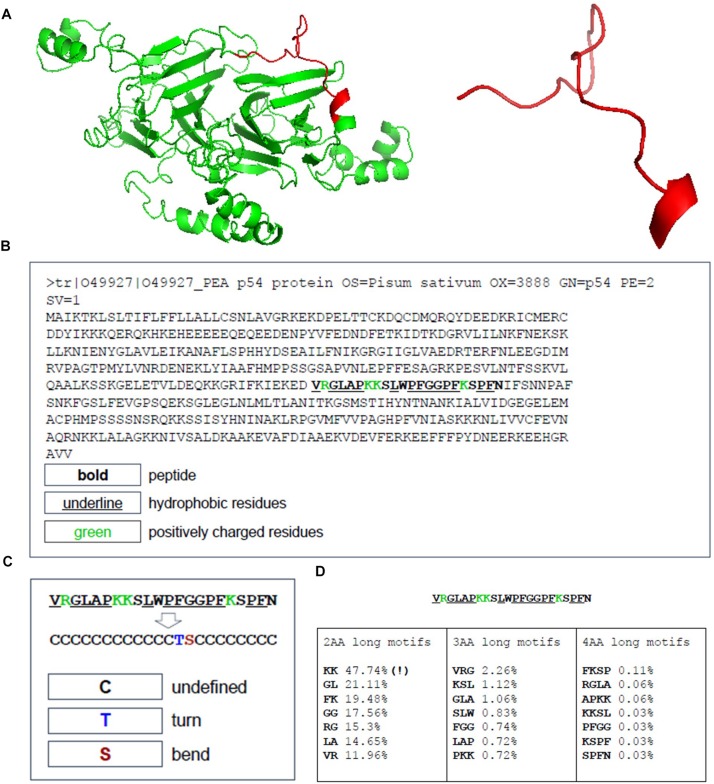
Sequence Location, structure and antimicrobial motifs of NuriPep 1653 derived from P54 *Pisum sativum* protein. The sequence structure and folding pattern as predicted by I-TASSER is shown in **(A)**. The sequence of the storage reservoir P54 protein with the location of NuriPep 1653 highlighted in bold, hydrophobic residues underlined and positive resides in green **(B)**. The peptide conformation is shown in **(C)** as determined by SCRATCH-1D. The most recurrent exact motifs of length 2, 3, and 4 amino acids within NuriPep 1653 and occurring in 3496 curated antimicrobial peptides are shown in **(D)**.

The peptide discovery approach used in this study is outlined in Figure 1. By using the top 20 features identified as important in conferring antimicrobial activity to rank the sequences identified from the MS data of hydrolyzed *P. sativum* protein material, NuriPep 1653 was identified as the top candidate for further investigation. Figure 2 is a t-distributed Stochastic Neighbour Embedding (t-SNE) plot representing all the peptides used in the study; positive and negative data sets obtained from CAMP and Uniprot (see text footnote 2) databases, respectively; MS data obtained by hydrolysis of *P. sativum*; and top scoring peptides from the feature ranking. Clusters in the data can be observed which may spear based on similar structures and characteristics e.g., negative peptides in co-ordinates (Gray −65, −15), and positive peptides in co-ordinates (Yellow −25, 45). The ∼4000 peptides identified by MS were wide spread across the plot (Blue) as were the 100 top ranked peptides (Red). NuriPep 1653 is represented by a pink star at (−38, −12). NuriPep 1653 (VRGLAPKKSLWPFGGPFKSPFN) is a linear 22-mer peptide with a global positive charge (+4) as a result of 1 arginine and 3 lysine residues in the sequence. Based on the cationic and amphipathic characteristics, we can assume a membranolytic step being involved in the killing of *A. baumannii*. The structure of the protein and isolated peptide was predicted by Iterative Threading ASSEmbly Refinement (I-TASSER^3^) (Figure 3A). It identifies structural templates from the PDB by multiple threading approach, with full-length atomic models constructed by iterative template-based fragment assembly simulations. The bioactive peptide is located inside the non-antimicrobial, storage protein P54 from *P. sativum* (Figure 3B) and is predicted to have a mostly linear structure with a single turn and a bend at residue 13 and 14, respectively, (Figure 3C). The lack of cysteine residues contributes to the linearity of the sequence. Finally, motifs identified as common from a set of 3496 known AMPs are outlined in Figure 3D. The 2-amino acid residues KK located in position 7 and 8 of NuriPep 1653 appear in 33% of the curated AMPs. Glycine paired with leucine, glycine and arginine also features highly as commonly conserved motifs and therefore might be important in antimicrobial function.

### Antimicrobial Activity of NuriPep 1653 Against colRAB

The antimicrobial activity of NuriPep 1653, magainin 2 and colistin against colSAB and colRAB are summarized in Table 2. The activity of NuriPep 1653 against a range of Gram-negative and -positive bacteria are also shown in the table. Interestingly, the concentrations of NuriPep 1653 required to eliminate both colSAB and colRAB, were identical at 12 μg/mL. Similar results were observed with magainin 2, however, at concentrations four times higher than NuriPep 1653. The CE concentration of 8 μg/mL confirmed resistance to colistin according to the EUCAST guidelines (MIC ≤ 2 μg/mL) (The European Committee on Antimicrobial Susceptibility Testing [EUCAST], 2019). While the peptides likely share an initial membranolytic mechanism of action based on their cationic nature, the difference in activity observed between NuriPep 1653 and colistin against colSAB and colRAB suggests that a difference may exist in a subsequent bactericidal action inside the cell. In addition to its activity against *A. baumannii*, NuriPep 1653 displays activity against three other ESKAPE pathogens (*P. aeruginosa*, *K. pneumoniae*, and *E. aerogenes*) with CE concentrations ranging from 8 μg/mL – 400 μg/mL. No activity was reported against either of the Gram–positive bacteria. This may be due to the difference in the cell wall composition compared to Gram-negative strains.

**TABLE 2:** Complete elimination values of NuriPep 1653 against colSAB and colRAB as well as a range of Gram-negative and -positive pathogens.

**Strain**	**NuriPep 1653**	**Magainin 2**	**Colistin**
	
	**μg/mL**		
colSAB	12	50	0.25
colRAB	12	50	8^(R)^
*S. maltophilia*	64	NT	NT
*B. cepacia*	NI	NT	NT
*P. aeruginosa*	8	NT	NT
*K. pneumoniae*	400	NT	NT
*E. coli*	100	NT	NT
*S.* Typhimurium	100	NT	NT
*S. aureus*	NI	NT	NT
*L. monocytogenes*	NI	NT	NT
*E. aerogenes*	200	NT	NT

*Values of NuriPep 1653 required to induce bacterial clearance *via* the complete elimination method against colSAB, colRAB and a range of Gram-negative and -positive pathogens. Magainin 2 and colistin were used as controls for the *A. baumannii* strains used in this study. ^(R)^ = resistant as defined by EUCAST breakpoint guidelines as (MIC ≤ 2 μg/mL). NI = no inhibition at concentrations <800 μg/mL. NT = not tested. Values shown represent the average of three independent experiments on three independent days.*

### Kinetics of Antimicrobial Action Induced by NuriPep 1653 Against colSAB and colRAB

**FIGURE 4:**
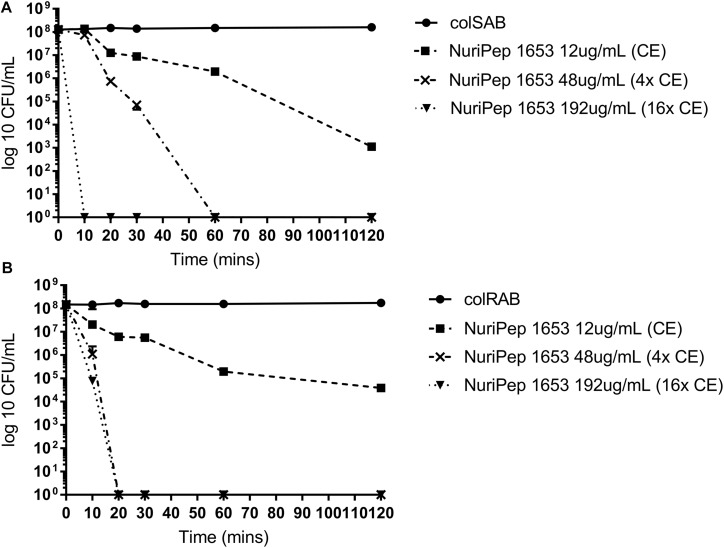
Killing kinetics of NuriPep 1653 against colRAB and colSAB. The killing activity of NuriPep 1653 against **(A)** colSAB and **(B)** colRAB was monitored for 120 min. Peptide treatments at 1× CE (12 μg/mL), 4× CE (48 μg/mL), and 16× CE (192 μg/mL) concentrations were tested. The CFU/mL was calculated at the timepoints 10, 20, 30, 60, and 120 min and was compared to the untreated control. Data represent the mean of three experiments performed in triplicate and is expressed as the mean ± standard deviation.

As shown in Figure 4, the rate of bacterial killing was concentration dependent in both colSAB and colRAB. At the CE concentration (12 μg/mL), between 10^4^ and 10^5^ CFU/mL were eliminated after 120 min for colSAB and colRAB, respectively. However, when the peptide concentration was increased to 48 μg/mL, total bacterial clearance (10^8^ CFU/mL) was observed after just 20 min in colRAB as opposed to 60 min in colSAB. At 16× the CE (192 μg/mL) rapid killing kinetics was achieved at 10 and 20 min for colSAB and colRAB, respectively. No regrowth was observed after 24 h indicating bactericidal activity as opposed to inhibitory action.

### Synergy Assessment of NuriPep 1653 and Colistin Against colRAB

**FIGURE 5:**
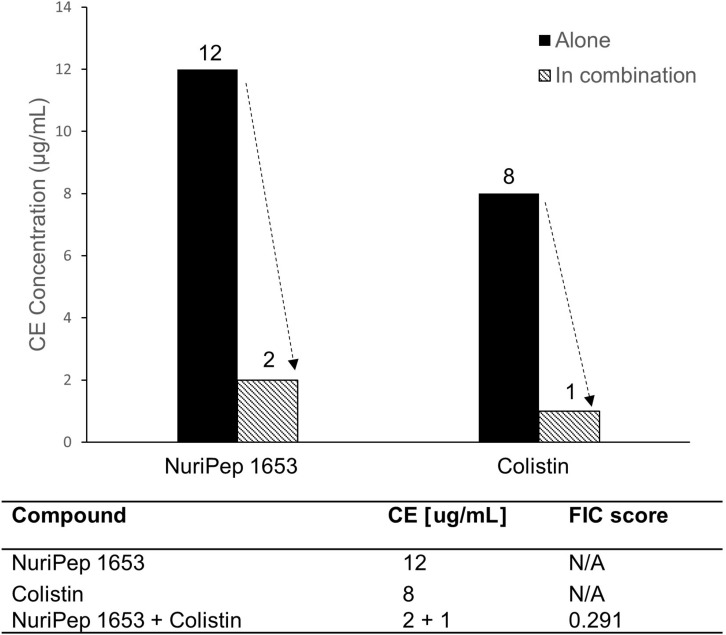
Assessment of synergistic interactions between NuriPep 1653 and colistin against colRAB. Changes in CE concentration observed with NuriPep 1653 and colistin against colRAB when tested alone *versus* in combination to determine synergistic effects. A FIC index score of 0.291 for both peptides combined indicates synergy.

In order to assess potential synergistic interactions between NuriPep 1653 and other Food and Drug Administration (FDA) approved AMPs, we combined the use of NuriPep 1653 with colistin, a last resort cationic lipopeptide. When NuriPep 1653 was combined with colistin, the CE concentrations could be reduced from 12 and 8 μg/mL, the concentrations previously determined to induce killing alone, respectively, to 2 and 1 μg/mL (Figure 5). Using the FIC index, this provides a score of 0.291, thus indicating a synergy between the two peptides. This is indicative that both peptides may kill *A. baumannii via* different mechanisms post initial disruption of the membrane based on the improved combined action.

### Significance of Electrostatic Interactions in the Initial Mechanism of Action of NuriPep 1653

**FIGURE 6:**
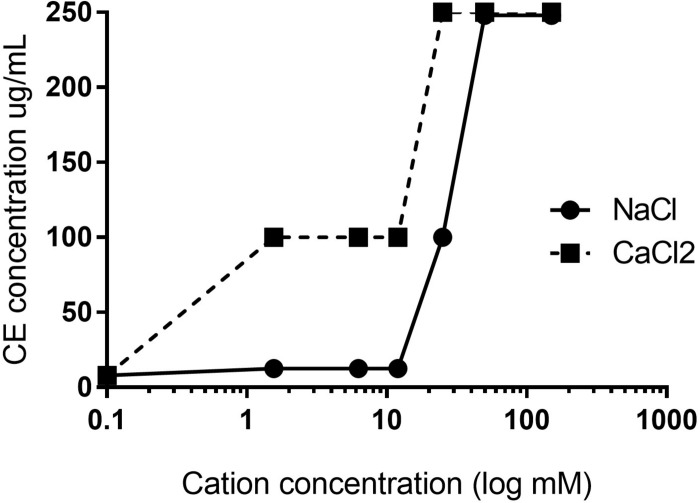
Electrostatic interactions and the impact of cations on the antimicrobial activity of NuriPep 1653. CE concentrations against colSAB were determined for NuriPep 1653 in the presence of increasing concentrations of NaCl or CaCl_2_. The initial CE under standard conditions was 12 μg/mL.

To validate the importance of electrostatic interactions in the mechanism of action of the cationic peptide NuriPep 1653, we assessed the activity of the peptide when pre-exposed to mono- and di-valent cations in the form of NaCl and CaCl_2_. Valence sensitivity was observed with the CE concentration increasing from 8 to 100 μg/mL in the presence of 12.5 mM of NaCl (Figure 6). Activity was completely lost when the peptide was exposed to either of the cationic buffers above 50 mM. These results confirm that disruption of electrostatic attractions induced a concentration and ion dependent inhibition which impacted peptide primary attachment and subsequent cellular disruption in colSAB.

### Cell Viability and Membrane Permeabilization of *A. baumannii* Post Treatment With NuriPep 1653

**FIGURE 7:**
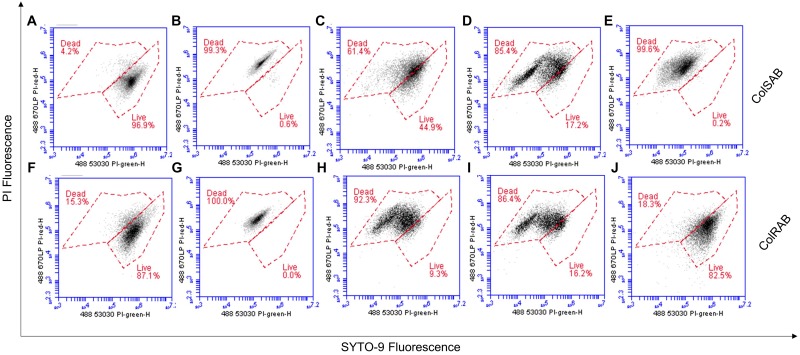
Analysis of the effects of NuriPep 1653 on the membrane integrity and viability of colRAB and colSAB. Quantification of viable *versus* non-viable cells represented by the uptake of fluorescent PI in **(A)** untreated colSAB; **(B)** heat inactivated colSAB at 95°C for 60 min; **(C)** colSAB treated with NuriPep 1653 at 12 μg/mL; **(D)** colSAB treated with magainin 2 at 12 μg/mL; **(E)** colSAB treated with colistin at 4 μg/mL; **(F)** untreated colRAB; **(G)** heat inactivated colRAB at 95°C for 60 min; **(H)** colRAB treated with NuriPep 1653 at 12 μg/mL; **(I)** colRAB treated with magainin 2 at 12 μg/mL; **(J)** colSAB treated with colistin at 4 μg/mL. A minimum of 50,000 single events were collected per sample. Data is displayed as scatter plots of PI fluorescence (*y*-axis) and SYTO-9 fluorescence (*x*-axis). When used alone, the SYTO9 stain generally labels all bacteria in a population. In contrast, PI penetrates only bacteria with damaged membranes, causing a reduction in the SYTO9 stain fluorescence when both dyes are present. Therefore, bacteria with intact cell membranes stain fluorescent green, whereas bacteria with damaged membranes stain fluorescent red. Optical densities of bacteria were optimized for cell events.

Cellular viability and membrane integrity of colSAB (Figures 7A–E) and colRAB (Figures 7F–J) pre- and post-treatment with NuriPep 1653 was analyzed by co-staining of the bacterial cells with two nucleic acid dyes (PI and SYTO-9) and measuring fluorescence. Untreated cells and heat-treated cells were used as measures of viability as observed by minimal and maximal PI fluorescence, respectively. ColSAB and colRAB cells exposed to NuriPep 1653 at 12 μg/mL are observed in Figures 7C,H, respectively, where 61 and 92% of cells are labeled as dead. Magainin 2 treated cells are shown in Figures 7D,I for the susceptible and resistant strains, where only ∼17 and 16% of cells were viable post-treatment, respectively. In Figure 7E, ∼99% of the colSAB cells treated with colistin showed PI uptake, indicating complete bacterial clearance as a consequence of membrane permeabilization, damage and death. Conversely, ∼82% of the colRAB cells remained viable further confirming the resistance profile and ineffectiveness of the lipopeptide treatment (Figure 7J). These results clearly indicate an increased permeability of *A. baumannii*, in both susceptible and resistant phenotypes, in response to NuriPep 1653 treatment.

### *In vitro* Development of Bacterial Resistance in Response to NuriPep 1653

**FIGURE 8:**
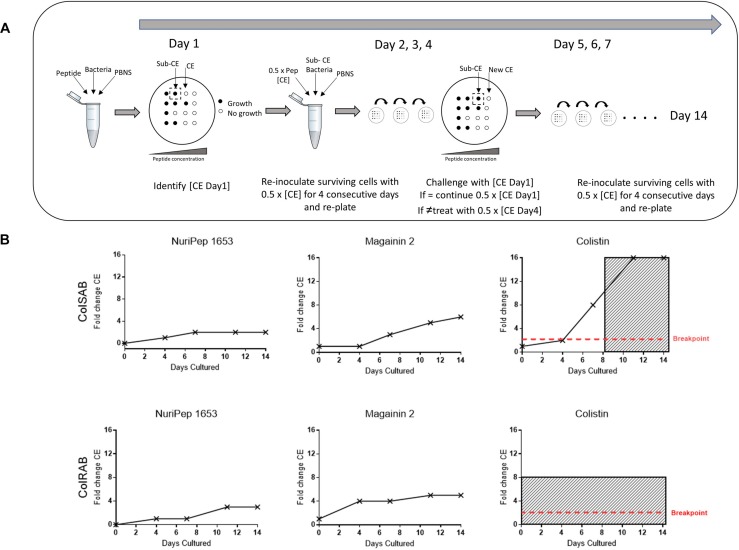
Assessment of the development of resistance to NuriPep 1653, magainin 2 and colistin in colRAB and colSAB over 14 sequential passages. A schematic representation of the resistance assay is shown in **(A)**. On day 0, bacterial cells were diluted in PBNS to 1 × 10^4^ cells/mL and were treated with either colistin, magainin 2 or NuriPep 1653 at a range of concentrations for 90 min. The CE concentration was determined on day 1 after 18 h. This was repeated throughout the experiment. On days 4, 7, 11, and 14, the bacteria were challenged with the previously determined CE concentration. Where the killing concentration was higher than that on day 0, the treatment for the following 3 days was increased to one half of the new CE concentration. The CE concentrations represented as a fold change over 14 days during repeated exposure of colSAB with sub inhibitory doses of NuriPep 1653, magainin 2 and colistin from left to right are shown in the top row **(B)** and again against colRAB in the bottom row. The development of resistance was defined as >10-fold the CE and is shown as shaded areas in the graphs (Deslouches et al., 2015). Colistin resistance in ColRAB was previously determined (Machado et al., 2018) and confirmed by the EUCAST breakpoints and is represented as resistant from day 0.

ColSAB and colRAB were both passaged repeatedly in the presence of either NuriPep 1653, magainin 2 and colistin at 50% of the CE value to explore the development of resistance. This concentration was insufficient to induce killing but the continual exposure allows for the selection of mutants that may exist or develop in a population, which can eventually persist as a resistant population. A schematic overview of the experimental procedure is shown in Figure 8A and the fold change observed in CE concentration over 14 days in Figure 8B. When NuriPep 1653 and colistin treatment in colSAB are compared, a 2-fold *versus* a 6-fold increase in the CE concentrations were observed, respectively, over 14 days. Interestingly, the greatest increase (16-fold) was seen in colistin treated colSAB. Here, the cells developed resistance after 8 days, where resistance was defined as >10-fold the original CE concentration (Deslouches et al., 2015). ColRAB cells remained susceptible to NuriPep 1653 over 14 days. The trend observed in magainin 2 treated cells suggests a slow tolerization and adaptation of cells over time. As colRAB was already identified as resistant to colistin by the EUCAST breakpoints (The European Committee on Antimicrobial Susceptibility Testing [EUCAST], 2019), the development of resistance through a fold change in CE was not determined and was represented as resistant from day 0 in Figure 8. NuriPep 1653 and colistin treated cells behave differently over the 14 days. The initially susceptible cells develop resistance to colistin just over half way through the time course. The CE concentration of NuriPep 1653 is identical in both colRAB and colSAB and neither develop resistance over 14 days exposure. These contrasting responses suggest different mechanisms of action, however, further experiments are needed to explore the details of this.

### Effects of NuriPep 1653 on the Viability of Differentiated Human Macrophages

**FIGURE 9:**
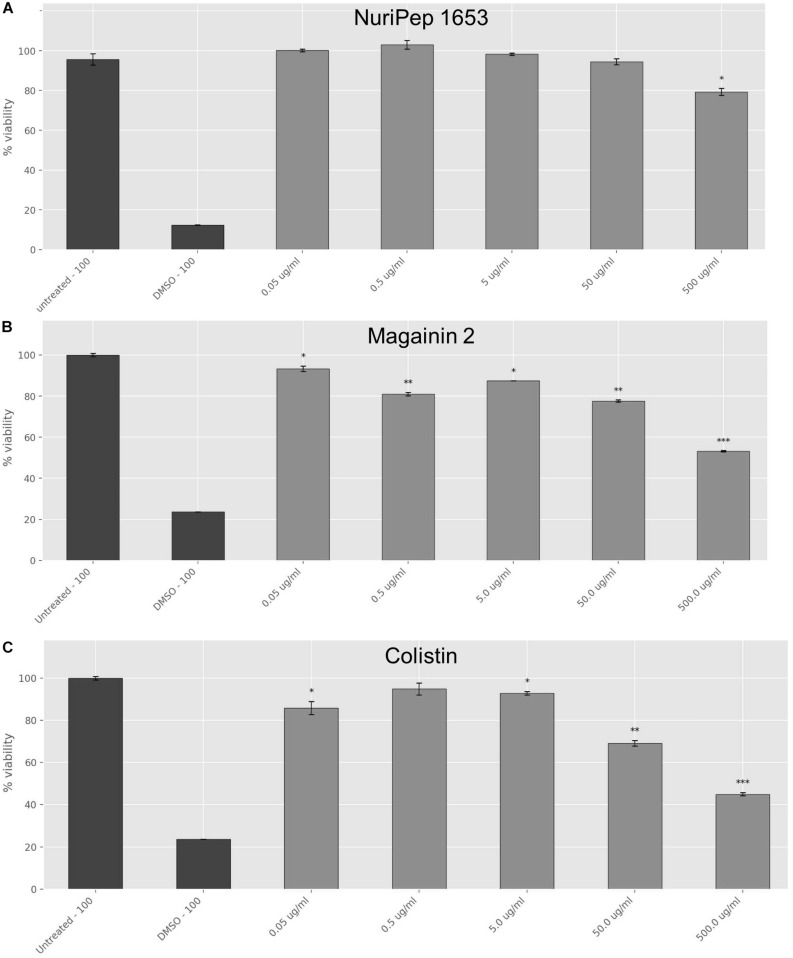
Effects of NuriPep 1653 on the viability of differentiated human macrophages. Cells were differentiated with 10 mM PMA for 72 h into macrophages before treatment with the peptide for 24 h. Cell viability was assessed by MTT assay as per the manufacturers guidelines. Values correspond to concentrations of **(A)** NuriPep 1653, **(B)** magainin 2 and **(C)** colistin tested at a range from 0.5–500 μg/mL. DMSO 100% and untreated cells served as controls. Data represent the mean of three experiments performed in triplicate and is expressed as the mean ± standard deviation. Statistical analysis was performed using a Student’s *t*-test where ^∗^*p* ≤ 0.05, ^∗∗^*p* ≤ 0.01, and ^∗∗∗^*p* ≤ 0.001.

Toxicity studies were carried out to determine the concentration dependent toxicity profile of the peptide in differentiated human macrophages and compare this to the bactericidal concentrations *in vitro*. When the antimicrobial activity assays were performed in RPMI, the CE concentration against colRAB was increased to >200 μg/mL (Supplementary Table S2), therefore, this media interfered with the peptide antimicrobial activity. At the moment we are unaware whether this interference is due to a disrupted peptide conformation or due to interaction of the peptide with any of the components found in the RPMI media and the results presented for the toxicity should be considered in the context of these results. As shown in Figure 9, no significant adverse impact on cellular viability was observed with concentrations of NuriPep 1653 as high as 50 μg/mL, however, a 24% decrease in cell viability was observed at 500 μg/mL (*p* ≤ 0.05). In the case of magainin 2, exposure of the cells to 50 μg/mL caused a decrease of 22% in viability (*p* ≤ 0.01). Contrastingly, at just 0.05 μg/mL of colistin, 12% of cells were killed (*p* ≤ 0.05). Furthermore, less than 50% of cells survived the highest treatment of 500 μg/mL (*p* ≤ 0.001). The results revealed that the toxic concentration of NuriPep 1653 is over 40 times higher than what is required for antimicrobial action, indicating a good therapeutic index or ratio of the toxic concentration compared to the therapeutic dose. Contrastingly, the toxic concentration for colistin is just 6.8 times higher than the active concentration against colRAB. NuriPep 1653 differs from the other two peptides in that it was identified as a naturally occurring sequence within a region of a *P. sativum* protein. It may display a better safety profile compared to the other two peptides as it is not produced as a secondary metabolite and due to its natural, edible source material. As mentioned above, these assays were conducted in RPMI media to give an initial idea of the toxic effects of the peptide, however, a more accurate evaluation of this would be to perform toxicity studies in a murine *in vivo* model over a specific time course. Additionally, when applied to sheep RBCs, neither NuriPep 1653, magainin 2 nor colistin were shown to induce significant hemolysis at concentrations as high as 500 μg/mL (data not shown).

## Discussion

The disbalance between the rate of bacterial resistance and discovery of anti-infective countermeasures is spiraling out of control (O’Neill, 2015). While carbapenem resistant *A. baumannii* is listed by the WHO as a “critical priority” pathogen, Qureshi et al. (2015) report the complex resistance mechanisms and surging prevalence of colistin resistance emerging in *A. baumannii* strains isolated from hospital settings (Ahmed et al., 2016). ColRAB was included in this study on this basis where remaining treatment options have been completely depleted. While cationic linear peptides have been proposed by many researchers as having significant potential as new anti-infective drugs, few have been successful based on toxicity, poor commercial viability and inferior activity when compared to antibiotics (Ge et al., 1999; Baltzar and Brown, 2011; Deslouches et al., 2015). In this work, we aimed to go beyond the realms of secondary metabolites and explore if AMPs can be identified within regions of non-antimicrobial proteins.

Embedded peptides are usually inactive in the parent protein but once proteolytically released, exert potent bioactivities (Mine et al., 2004; Korhonen and Pihlanto, 2006). The reasons behind the existence of these sequences within larger proteins remains unknown. It may be that the peptides exist to confer health benefits once ingested. Since the nineteenth century, it was understood that milk, once consumed, played a vital role in neonatal protection and that the beneficial components were potentiated through digestion (Fokker, 1890; Ehrlich, 1892; Fadaei, 2012). Interestingly, this concept and the evolutionary importance of these sub-peptide sequences is virtually unexplored in plants. It is conceivable to think, that peptides get released by natural cleavage at a given stage in the life cycle of the plant before being further broken down into individual amino acid building blocks. The functionalities of many of the intermediate breakdown products remain undetermined. We explore legumes and their bioactive breakdown protein products, expanding knowledge beyond the well studied scope of milk.

NuriPep 1653, a novel natural non-toxic peptide was identified from within a region of the P54 protein in *P. sativum* and was released post enzymatic digestion of the protein. To the best of our knowledge, the sequence of the peptide has not been previously reported. NuriPep 1653 features highly in pre-established antimicrobial characteristics and displayed potent bactericidal action against PDR *A. baumannii* at 12 μg/mL *in vitro*. This peptide was also shown to possess activity against 3 other ESKAPE pathogens (*P. aeruginosa*, *K. pneumoniae*, and *E. aerogenes*) as well as *S. maltophilia* which is prevalent in cystic fibrosis and respiratory disease. It should be noted that the majority of peptides with antimicrobial activity in line with that observed for NuriPep 1653 against a PDR strain, are either completely synthetic creations, or engineered versions of natural compounds (Deslouches et al., 2015). NuriPep 1653 is completely unmodified, linear, shorter than the majority of known plant peptides and naturally occurring in a phyto-protein. While in this work we highlight just one peptide with a focus on its potential pharmaceutical application, the mere existence of NuriPep 1653 highlights the possible presence of many other AMPs of the same nature. This approach vastly extends the potential of peptide discovery from plants beyond those which are ribosomally derived or produced as metabolites.

Common to other AMPs, the activity of NuriPep 1653 was disrupted by salt sensitivity (Klüver et al., 2006; Holthaus et al., 2016), however, the peptide showed thermostability when heated at 95°C for 1 h prior to bacterial challenge (Supplementary Figure S2). Further details of the assay are outlined in Supplementary Methods and Supplementary Results. Cationic, amphipathic peptides rely on electrostatic interactions for bacterial attachment or insertion as an initial step in their mechanism (Menzel et al., 2016). We confirmed this by observing a concentration- and valence- dependent inhibition of the bioactivity of NuriPep 1653 once solubilized in buffers with varying anionic strength. Considering this, a general lack of toxicity of AMPs to eukaryotic cells is assumed based on their zwitterionic phospholipids membrane, which have minimal electrostatic potential (Yeaman and Yount, 2003). We determined the cytotoxic concentrations of NuriPep 1653 in THP-1 cells which was 40 times higher than that required to eliminate bacterial growth *in vitro*. The toxic concentration of colistin was in line with that reported in literature where a significant reduction in cell viability occurred at 0.05 μg/mL (Marr et al., 2006; Uhlig et al., 2014). The improved safety profile of NuriPep 1653 over other AMPs may be based on its natural occurrence, natural edible source and lack of non-natural amino acids.

Following membrane binding, PI was used as an indicator of membrane permeability to assess if NuriPep 1653 exerts it’s action through a membrane active mechanism, commonly observed among AMPs (Bahar and Ren, 2013; Wang et al., 2015). The cellular integrity of both colRAB and colSAB were affected with 92 and 61% of cells labeled as non-viable post peptide treatment. Mirroring the findings from the killing kinetics, NuriPep 1653 showed improved activity in the PDR isolate over colSAB. Lázár et al. (2018) highlighted that increased collateral sensitivity to AMPs in MDR strains was stimulated by regulatory changes affecting the lipopolysaccharide (LPS) composition of the outer membrane which strengthened attachment and slowed down *de novo* evolution of resistance. From this we can assume that NuriPep 1653 may kill colRAB faster as developing resistance to antibiotics might have induced a high frequency collateral sensitivity to AMPs.

SEM images (Supplementary Figure S1) further highlight the effects of NuriPep 1653 on the permeabilization of the bacterial outer membrane. Control cells are shown in Supplementary Figures S1A,C. Similar ultrastructural degenerative aspects were observed with colSAB (Supplementary Figure S1B) and colRAB (Supplementary Figure S1D), however, in the latter, an elongated morphology was observed as the cells attempt to increase their surface area to dilute the potency of the peptide on the membrane. Complete lysis and cell debris were seen when cells were exposed to a higher concentration of the peptide.

Taken together, the above results indicate that NuriPep 1653 performs similarly to other cationic AMPs as regards its initial antimicrobial mechanism (Bahar and Ren, 2013). However, the synergistic effects observed against colRAB when the peptide was combined with colistin are suggestive of different actions post membrane disruption. CE concentration reductions of 6 and 8-fold were observed for NuriPep 1653 and colistin, respectively, indicating compound potentiation. This was reinforced in the induction of resistant phenotypes experiment. ColSAB remained sensitive to both NuriPep 1653 and magainin 2 throughout the 14 days as active concentrations remained below the threshold for resistance (fold change CE > 10). These findings are in line with other literature where unsuccessful attempts to generate resistance in several bacterial species through 14 repeated passages of pexiganan were reported (Ge et al., 1999). Similarly, in another study, *P. aeruginosa* was shown to develop resistance to colistin almost twice as fast as to two *de novo* engineered peptides, WLBU2 and WR12 and seven times faster to rifampicin (Deslouches et al., 2015). The differences in the generation of mutants by the eighth day between colistin and NuriPep 1653 treated cells eludes to a distinct mechanism of action between these two peptides post bacterial attachment. The limited propensity for inducing resistance adds to the attractiveness of NuriPep 1653 as a novel anti-infective or adjuvant compound capable of reverting resistant phenotypes.

The lack of observed development of resistance and action of NuriPep 1653 against clinically relevant pathogens positions NuriPep 1653 as an appealing candidate which warrants consideration for development and further testing on a larger cohort of PDR and XDR isolates. Therapeutic areas of particular interest may include cystic fibrosis and lung/skin infections given the results against the strains highlighted in Table 2. Future perspectives for NuriPep 1653 include increasing the salt resistance profile of the peptide. This may be investigated in future work through replacing tryptophan or histidine residues with the bulky amino acids β-naphthylalanine and β-(4,4′-biphenyl)alanine, as described by Yu et al. (2011) for their novel peptide P-113. Furthermore, a deeper exploration into activity, protease resistance, bioavailability and optimal delivery strategies in murine models will also be investigated in the future.

## Patent Number

The sequence disclosed in this paper is covered in a patent (Submission Number: 7783813; Application Number: EP19192678.1).

## Data Availability

The raw data supporting the conclusions of this manuscript will be made available by the authors, without undue reservation, to any qualified researcher.

## Author Contributions

NM wrote the manuscript, and designed and performed the majority of experiments. AZ contributed to the interpretation of the results and provided critical feedback and helped to shape the research. GJ performed the data mining and bioinformatic analysis. AK performed the *ex vivo* experiments. NK devised the project, the main conceptual ideas, and theoretical framework related to the data mining of the peptide. MM was involved in the design of the experiments, the approach regarding the mode of action of the peptide, and interpretation of the results. All authors reviewed the manuscript.

## Conflict of Interest Statement

AZ, GJ, AK, and NK were employed by company Nuritas Limited. The remaining authors declare that the research was conducted in the absence of any commercial or financial relationships that could be construed as a potential conflict of interest.

## Footnotes

1 http://www.camp.bicnirrh.res.in/

2 https://www.uniprot.org/

3 https://zhanglab.ccmb.med.umich.edu/
