# Supplementary material for: Unlocking NuriPep 1653 From Common Pea Protein: A Potent Antimicrobial Peptide to Tackle a Pan-Drug Resistant Acinetobacter baumannii
Source: Front Microbiol. 2019 Sep 18;10:2086. doi: 10.3389/fmicb.2019.02086 (PMC6759681; doi:10.3389/fmicb.2019.02086)
Supplement: Supplementary file 1 [file Table_1.docx]

**Supplemental Table 1: Ranked list of top 20 sequence physiochemical properties in conferring antimicrobial activity to peptides**

| Feature ranking | AAIndex identifier | AAIndex identifier feature | Feature group |
| --- | --- | --- | --- |
| 1 | KLEP840101 | net charge | Charge |
| 2 | QIAN880113 | Weights for alpha-helix, pos 6 | Structure |
| 3 | QIAN880127 | Weights for coil at the window position of -6 | Structure |
| 4 | QIAN880101 | Weights for alpha-helix, pos -6 | Structure |
| 5 | FAUJ880111 | positive charge | Charge |
| 6 | SUEM840102 | Zimm-Bragg parameter sigma - helix-coil transition | Structure |
| 7 | WILM950101 | Hydrophobicity coefficient in RP-HPLC, C18 with 0.1%TFA/MeCN/H2O | Hydrophobicity |
| 8 | VENT840101 | bitterness |  |
| 9 | ARGP820103 | Membrane-buried preference parameters | Hydrophobicity |
| 10 | BROC820102 | Retention coefficient in HFBA | Hydrophobicity |
| 11 | WILM950102 | Hydrophobicity coefficient in RP-HPLC, C8 with 0.1%TFA/MeCN/H2O | Hydrophobicity |
| 12 | NAKH920108 | AA composition of MEM of multi-spanning proteins | Cytoplasmic vs extracellular AA composition of the membrane |
| 13 | NAKH900110 | Normalized composition of membrane proteins | Cytoplasmic vs extracellular AA composition of the membrane |
| 14 | QIAN880130 | Weights for coil at the window position of -3 | Structure |
| 15 | MEEJ800101 | Retention coefficient in HPLC, pH7.4 | Hydrophobicity |
| 16 | ROBB760104 | Information measure for C-terminal helix | Structure |
| 17 | NAKH900112 | Transmembrane regions of mt-proteins | Cytoplasmic vs extracellular AA composition of the membrane |
| 18 | ROBB760107 | Information measure for extended without H-bond | Structure |
| 19 | GARJ730101 | Partition coefficient | Hydrophobicity |
| 20 | PONP800107 | Accessibility reduction ratio | Hydrophobicity |

**Legend:** Sequence physiochemical properties obtained from the AAIndex and ranked according to the discovery pipeline in Figure 1. Scores are normalised by peptide length and ranked backwards from 1 – 20 with number 1 being the top ranked. These features we used in the ranking of the mass spectrometry peptide output according to most likely to possess antimicrobial activity. The features can be grouped broadly into 4 categories: charge, structure, hydrophobicity and amino acid composition.
