# Supplementary material for: Unlocking NuriPep 1653 From Common Pea Protein: A Potent Antimicrobial Peptide to Tackle a Pan-Drug Resistant Acinetobacter baumannii
Source: Front Microbiol. 2019 Sep 18;10:2086. doi: 10.3389/fmicb.2019.02086 (PMC6759681; doi:10.3389/fmicb.2019.02086)
Supplement: Supplementary file 2 [file Table_2.docx]

**Supplemental Table 2: Complete Elimination values of NuriPep 1653, Magainin 2 and Colistin against colRAB in various media**

| Media | NuriPep 1653 | | | Magainin 2 | |  | | Colistin |
| --- | --- | --- | --- | --- | --- | --- | --- | --- |
|  | |  | | | | | | |
| Phosphate Buffer (without salt) | | 12 | 50 | |  | | 8 | |
| Phosphate Buffer (with salt) | | >200 | 100 | |  | | 8 | |
| Mueller Hinton cation adjusted | | >400 | 100 | |  | | 8 | |
| Nutrient Broth | | >400 | NT | |  | | NT | |
| Brain Heart Infusion Broth | | >400 | NT | |  | | NT | |
| RPMI media | | >200 | NT | |  | | NT | |

**Legend:** Values of NuriPep 1653, Magainin 2 and Colistin required to induce bacterial clearance *via* the complete elimination method using various media against colRAB. RPMI - Roswell Park Memorial Institute medium; NT = Not tested. Values shown represent the average of three independent experiments on three independent days.
