# Supplementary material for: Unlocking NuriPep 1653 From Common Pea Protein: A Potent Antimicrobial Peptide to Tackle a Pan-Drug Resistant Acinetobacter baumannii
Source: Front Microbiol. 2019 Sep 18;10:2086. doi: 10.3389/fmicb.2019.02086 (PMC6759681; doi:10.3389/fmicb.2019.02086)
Supplement: Supplementary file 3 [file Data_Sheet_1.docx]

**Supplemental Data**

**Unlocking NuriPep 1653 from common Pea Protein: a Potent Antimicrobial Peptide to Tackle a Pan-Drug resistant *Acinetobacter baumannii***

Mohan NM.^1,2^, Zorgani A.^2*^, Jalowicki G.^2^, Kerr A.^2^, Khaldi N.^2^ and Martins M.^1*^.

^1^Department of Microbiology, Moyne Institute of Preventive Medicine, School of Genetics and Microbiology, Trinity College Dublin, The University of Dublin, Dublin 2, Ireland.

^2^Nuritas limited, Joshua Dawson House,19B Dawson Street Dublin 2 B, Dublin, D02 RY95.

***Corresponding authors:**

Amine Zorgani, Joshua Dawson House,19B Dawson Street Dublin 2 B, Dublin, D02 RY95.

Marta Martins, Department of Microbiology, Moyne Institute of Preventive Medicine, School of Genetics and Microbiology, Trinity College Dublin, The University of Dublin, Dublin 2, Ireland; Phone: +353 1 896 1194; e-mail: mmartins@tcd.ie

**SUPPLEMENTAL METHODS**

***Thermostability Study:*** The thermostability of NuriPep 1653 was assessed as per the CE method described, however, prior to the bacterial challenge, the peptide was incubated at temperatures of 37, 75, 95 and 121°C.

**SUPPLEMENTAL RESULTS**

***Scanning electron microscopy (SEM)***

The structural damage and morphological changes induced in colSAB and colRAB after treatment with NuriPep 1653 at a sub lethal concentration using SEM are shown in Fig. S1. Untreated colSAB (Fig. S1A) and colRAB (Fig. S1C) display smooth, intact surfaces and active proliferation. The cells are numerous, appear healthy and viable which was confirmed by plating onto MHA and determining the CFU/mL before fixation with glutaraldehyde (data not shown). Conversely, NuriPep 1653 treated cells, shown in Fig. S1B and S1D exhibit major morphological changes such as roughening of the cell surface and distorted cell surface formation. The peptide treated colSAB cells (Fig. S1B) appear elongated compared to the control cells (Fig. S1A). This is likely an axial stress response induced by the peptide. The cells attempt to increase their cell surface area to dilute the potency of NuriPep 1653 on a site in the membrane. When a higher concentration of the peptide was used, no intact cells were observed and instead, substantial debris was seen indicating complete cell disruption and a loss of cellular integrity (data not shown).

***Temperature stability assessment of NuriPep 1653 across a range from 37 – 95°C***

The thermostability of NuriPep 1653 was evaluated in colSAB (Fig. S2). The peptide was shown to resist high temperatures and retain the same CE concentration (12 µg/mL) under both standard conditions at 37°C and after exposure to 95°C for 60 minutes. Only after incubation at 121°C was the peptide rendered inactive likely due to complete denaturation and breakdown of the sequence.

***Sequence physicochemical properties ranked as important in conferring antimicrobial activity to peptides in the discovery pipeline***

Supplementary Table 1 defines the AAIndex identifier string and description of the top 20 ranked features employed in the discovery pipeline (section 2.1) to sort the peptides identified by mass spectrometry according to predicted activity. The full list of 566 amino acid indices can be searched on the AAIndex^[[1]](#footnote-1)^. The 20 features can be grouped broadly into four groups: charge, structure, hydrophobicity and amino acid composition. Membrane interaction is critical to antimicrobial activity along with positive charge and hydrophobicity which allow a sequence to adopt a helical structure from a cytoplasmic coil structure.

1. https://www.genome.jp/aaindex/ [↑](#footnote-ref-1)
