# Supplementary material for: Unlocking NuriPep 1653 From Common Pea Protein: A Potent Antimicrobial Peptide to Tackle a Pan-Drug Resistant Acinetobacter baumannii
Source: Front Microbiol. 2019 Sep 18;10:2086. doi: 10.3389/fmicb.2019.02086 (PMC6759681; doi:10.3389/fmicb.2019.02086)
Supplement: Supplementary file 4 [file Data_Sheet_2.docx]

**Figure S1: Scanning electron micrographs of colSAB and colRAB post treatment with NuriPep 1653**

**Legend:** The images highlight the morphology of (A) Untreated colSAB; (B) NuriPep 1653 treated colSAB; (C) Untreated colRAB; (D) NuriPep 1653 treated colRAB. Cells were exposed to the peptide for 90 minutes before fixation. The treatment of *A. baumannii* with the peptide induced roughening of cell surface, elongation, disruption and debris while smooth cell surfaces were observed in cells without treatment.
