# Supplementary material for: Unlocking NuriPep 1653 From Common Pea Protein: A Potent Antimicrobial Peptide to Tackle a Pan-Drug Resistant Acinetobacter baumannii
Source: Front Microbiol. 2019 Sep 18;10:2086. doi: 10.3389/fmicb.2019.02086 (PMC6759681; doi:10.3389/fmicb.2019.02086)
Supplement: Supplementary file 5 [file Data_Sheet_3.docx]

**Figure S2. Thermostability of NuriPep 1653**

**Legend**: The CE concentrations of NuriPep 1653 against colSAB were determined when incubated at 35, 75, 95 or 121°C for 60 minutes prior to the bacterial challenge. Under standard conditions, assays were performed at room temperature ~ 21 ± 2°C.
