# Supplementary figures and images for: Unlocking NuriPep 1653 From Common Pea Protein: A Potent Antimicrobial Peptide to Tackle a Pan-Drug Resistant Acinetobacter baumannii
Source: Front Microbiol. 2019 Sep 18;10:2086. doi: 10.3389/fmicb.2019.02086 (PMC6759681; doi:10.3389/fmicb.2019.02086)

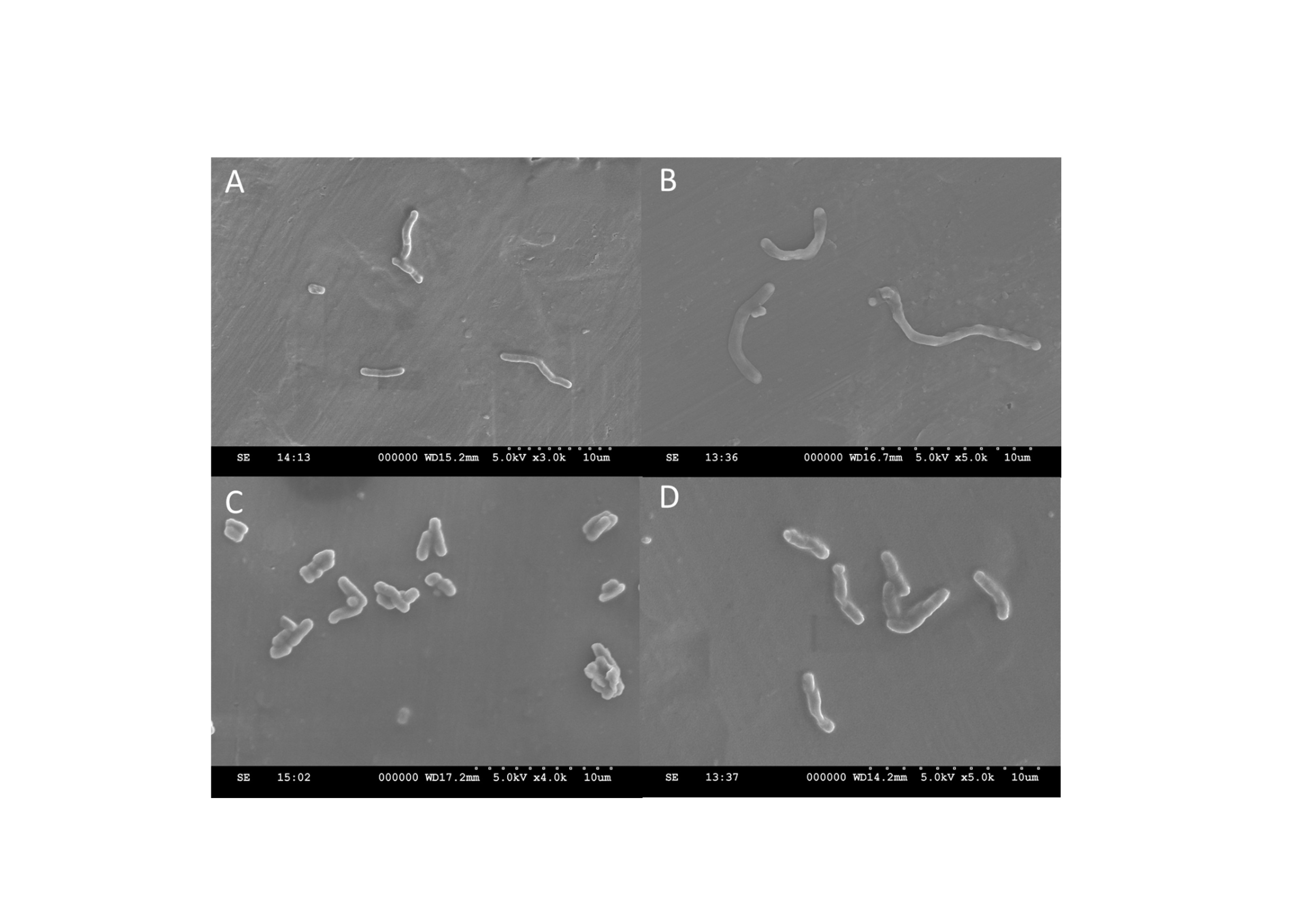

Supplement: Supplementary file 6 [file Image_1.jpg]

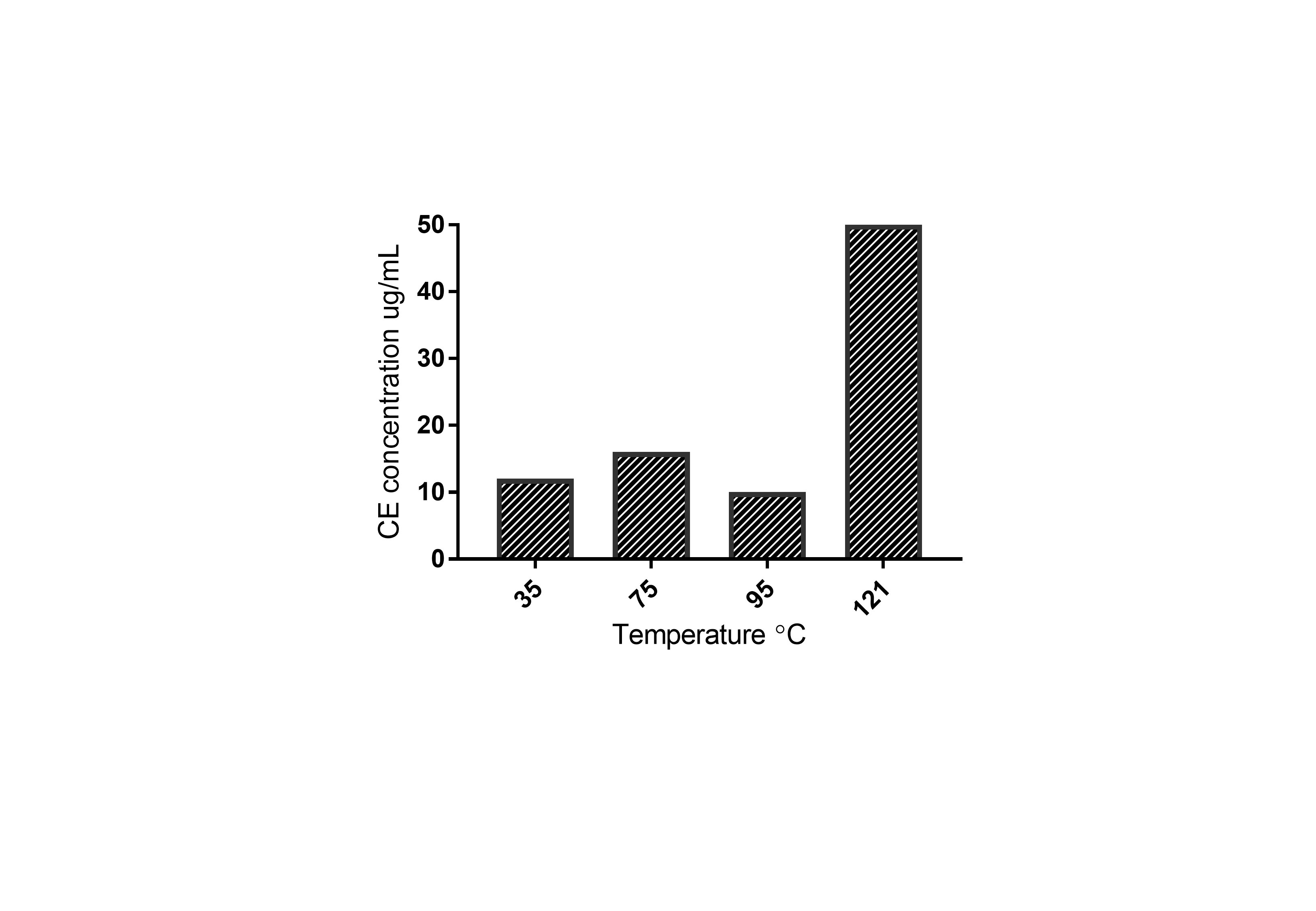

Supplement: Supplementary file 7 [file Image_2.jpg]
